# Engineered Root Bacteria Release Plant-Available Phosphate from Phytate

**DOI:** 10.1128/AEM.01210-19

**Published:** 2019-08-29

**Authors:** Christine N. Shulse, Mansi Chovatia, Carolyn Agosto, Gaoyan Wang, Matthew Hamilton, Samuel Deutsch, Yasuo Yoshikuni, Matthew J. Blow

**Affiliations:** a Department of Energy Joint Genome Institute, Walnut Creek, California, USA; Wageningen University

**Keywords:** *Pseudomonas*, synthetic biology, organic phosphorus, phytase, phytate, plant growth-promoting bacteria, root colonization

## Abstract

Microorganisms that release plant-available phosphate from natural soil phosphate stores may serve as biological alternatives to costly and environmentally damaging phosphate fertilizers. To explore this possibility, we engineered a collection of root bacteria to release plant-available orthophosphate from phytate, an abundant phosphate source in many soils. We identified 82 phylogenetically diverse phytase genes, refactored their sequences for optimal expression in *Proteobacteria*, and then synthesized and engineered them into the genomes of three root-colonizing bacteria. Liquid culture assays revealed 41 engineered strains with high levels of phytate hydrolysis. Among these, we identified 12 strains across three bacterial hosts that confer a growth advantage on the model plant Arabidopsis thaliana when phytate is the sole phosphate source. These data demonstrate that DNA synthesis approaches can be used to generate plant-associated strains with novel phosphate-solubilizing capabilities.

**IMPORTANCE** Phosphate fertilizers are essential for high-yield agriculture yet are costly and environmentally damaging. Microbes that release soluble phosphate from naturally occurring sources in the soil are appealing, as they may reduce the need for such fertilizers. In this study, we used synthetic biology approaches to create a collection of engineered root-associated microbes with the ability to release phosphate from phytate. We demonstrate that these strains improve plant growth under phosphorus-limited conditions. This represents a first step in the development of phosphate-mining bacteria for future use in crop systems.

## Abstract (precis)

Phosphate fertilizers are essential for high-yield agriculture yet are costly and environmentally damaging. Microbes that release soluble phosphate from naturally occurring sources in the soil are appealing, as they may reduce the need for such fertilizers. In this study, we used synthetic biology approaches to create a collection of engineered root-associated microbes with the ability to release phosphate from phytate. We demonstrate that these strains improve plant growth under phosphorus-limited conditions. This represents a first step in the development of phosphate-mining bacteria for future use in crop systems.

## INTRODUCTION

Phosphorus is an essential and limiting nutrient for plant growth and is obtained by uptake of orthophosphate (P_i_) through the roots. Phosphate fertilizers sustain high-yield agriculture but are nonrenewable, are from politically sensitive regions of the world, and pollute aquatic environments (1). Alternatives to phosphate-based fertilizers are needed to alleviate these problems.

One potential source of phosphate is represented by the abundant but recalcitrant forms of phosphate already present in the soil (2). Soil inorganic phosphates include iron, calcium, and aluminum precipitates of phosphate and phosphate adsorbed to the surface of soil minerals. Organic phosphates, derived from soil biomass (plant, microbial, and metazoan), are chemically diverse but dominated by phytic acid, primarily in the salt form, referred to as phytate (*myo*-inositol 1,2,3,4,5,6-hexakisphosphate [IP6]) (3). Organic phosphates such as phytate can be further bound by soil particles through adsorption-desorption reactions (4, 5). It has been estimated that accumulated phosphorus in agricultural soils may be sufficient to sustain high-yield agriculture for many decades (6). However, only ∼0.1% is in a form available to plants (7). The remainder must first be converted to soluble phosphate.

Plants and soil microorganisms have evolved diverse mechanisms to obtain phosphate from existing sources in the soil. These include expression of phosphatase and phytase enzymes to release P_i_ from organic phosphates (8) and exudation of organic acids and siderophores to solubilize inorganic phosphate (9–11). However, most natural plant and microbial communities are unable to produce high yields in the absence of added phosphate fertilizers. One approach is engineering plants to metabolize alternative forms of phosphate (12, 13). However, each crop species must be engineered individually and must be labeled as a genetically modified organism. An alternative is to develop phosphate-solubilizing soil microorganisms that grow in the vicinity of plant roots and release plant-available phosphate (14, 15). Bacteria are more amenable to engineering at scale and are potentially applicable to a broad range of plant species and environments. Furthermore, different bacteria would be expected to be differently successful at colonizing distinct crops and distinct soil environments. Therefore, it may be useful to develop several engineered bacterial strains, which could be used in a customized manner depending on crop and environment.

Previous studies exploring the use of phosphate-solubilizing microbes have largely focused on natural phosphate-solubilizing plant growth-promoting bacteria (PGPB) (e.g., see references 16–19). Other studies have introduced P solubilization genes into plant-associated hosts (20–22), but these typically rely on plasmids which are genetically unstable (23). Genomic integrants have been created for a single phosphate solubilization pathway (24), but the resulting strains were not tested for activity in plants.

Here, we used a combinatorial synthetic biology-based approach to generate a collection of plant-associated bacteria capable of efficient phytate hydrolysis. We engineered 82 biochemically diverse phytase enzymes directly into the genomes of three bacterial hosts and demonstrated that the resulting strains are highly efficient hydrolyzers of phytate in liquid culture. Inoculation of Arabidopsis thaliana with several of these strains results in significantly improved plant growth under soilless conditions.

## RESULTS

### Generation of transgenic bacterial strains overexpressing a diversity of phytases.

**FIG 1:**
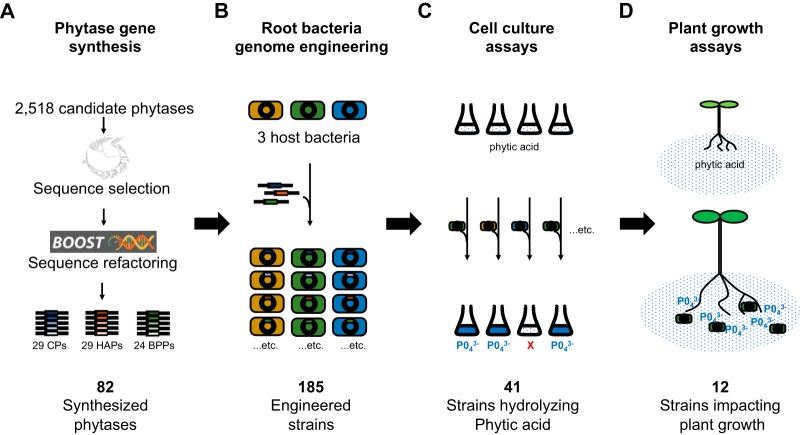
Study overview. (A) Diverse phytases were selected following a search of the Joint Genome Institute’s Integrated Microbial Genomes and Microbiomes (IMG/M) database. Figure includes the three classes of phytase enzymes in microbes: cysteine phytases (CPs), histidine acid phytases (HAPs), and beta-propeller phytases (BPPs). (B) Genes were optimized for expression in *Proteobacteria*, synthesized, and engineered into three bacterial hosts. (C) Engineered strains were evaluated *in vitro* for their ability to hydrolyze phytate and release P_i_. (D) *Arabidopsis* was inoculated with the best-performing strains and monitored for improvements in fresh and dry weights and rosette size.

We aimed to create a collection of plant-associated P_i_-releasing bacteria by engineering diverse P_i_-liberating enzymes into the genomes of multiple host bacteria (Fig. 1). Combining multiple hosts with diverse enzymes should result in a collection of strains with potential activity under a broader range of environmental conditions than any single host gene combinations.

For bacterial hosts, we chose the proteobacteria Pseudomonas simiae WCS417r, *Ralstonia* sp. strain UNC404CL21Col, and Pseudomonas putida KT2440. These bacteria are known to be stably associated with plant roots (25, 26), and each was previously engineered to contain a “landing pad” sequence that facilitates permanent cloning into the genome versus unstable cloning into plasmids (27), creating strains *P. simiae* WCS417r:pw17, *Ralstonia* sp. UNC404CL21Col:SB352_2, and P. putida KT2440:SB98_8.1. Furthermore, all three strains were shown to release P_i_ from tricalcium phosphate after 1 to 3 days of growth (see Fig. S1 in the supplemental material), presumably through the production of organic acids. This is important, as organic acids increase phosphate mobilization in soils by competing with phosphate for positively charged binding sites on soil colloids (28).

A broad suite of genes are potentially involved in P_i_ mineralization in soils (see Table S1). We selected phytases, as these catalyze the hydrolysis of phytate, one of the most abundant phosphorus-containing molecules in soil (3). There are three major classes of enzyme with demonstrated phytase activity in microorganisms: (i) histidine acid phosphatase (HAP), (ii) β-propeller phytase (BPP), and (iii) cysteine phosphatase (CP) (29). A search for phytase genes among all available microbial genomes and environmental metagenomes in the Integrated Microbial Genomes database (37,985 genomes and 6674 metagenomes as of 1 July 2015 [30, 31]) revealed a total of 2,518 potential phytases across these three classes. Using a phylogenetic sampling approach (32), we selected 82 genes with the maximum degree of sequence diversity. This set of enzymes included 29 HAPs, 24 BPPs, and 29 CPs, with representative sequences from across eight bacterial phyla and 7 sequences of unknown origin from metagenomic data sets (see Table S2). Of the three bacterial hosts, *P. simiae* WCS417r, *Ralstonia* sp. UNC404CL21Col, and P. putida KT2440, only *P. simiae* WCS417r contains a putative phytase gene (a BPP) (see Note S1).

The selected phytase gene sequences were refactored *in silico* for optimal expression in *Proteobacteria* (33). Briefly, DNA sequences were redesigned to reflect the nucleotide sequence composition and regulatory sequences of the *Pseudomonas* hosts while maintaining the original amino acid sequences. We then synthesized these genes, cloned them into high-expression isopropyl-β-d-thiogalactopyranoside (IPTG)-inducible expression cassettes, and sequence verified them using an established DNA synthesis pipeline (34). Finally, we transferred all 82 sequences into the genomes of *P. simiae* and *Ralstonia* sp. using conjugation and reconfirmed the sequences of the final engineered strains. We additionally transferred 21 sequences into Pseudomonas putida. In total, we generated 185 phytase-containing strains (Table S3).

### Engineered strains hydrolyze phytate in liquid culture.

**FIG 2:**
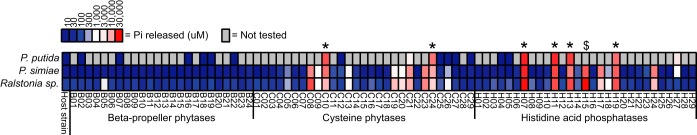
Phytase activity of engineered strains in liquid culture. P_i_ levels in culture supernatant where phytate is the only phosphate source at 10 days for each construct expressed in P. putida KT2440, *P. simiae* WCS417r, and *Ralstonia* sp. PGPB with engineered phytases are screened for the ability to release P_i_ from phytate using the malachite green-based QuantiChrom phosphate assay kit (BioAssay Systems). Each category of enzyme is ordered by amino acid length, from shortest to longest. Gray space indicates no measurement taken. *, strains used in plant assays; $, E. coli AppA included as positive control.

We next determined the ability of engineered strains to solubilize phytate in liquid culture. Strains engineered with a landing pad sequence but no phytase gene released no or negligible P_i_ from phytate at 10 days (*P. simiae* WCS417r:pw17, 8 μM; P. putida KT2440:SB98_8.1, 0 μM; *Ralstonia* sp. UNC404CL21Col:SB352_2, 84 μM) (Fig. 2). In contrast, 17/82 *P. simiae* phytase-harboring strains, 16/82 *Ralstonia* sp. phytase-harboring strains, and 8/21 tested P. putida phytase-harboring strains released high levels of P_i_ (>500 μM) after 10 days (Fig. 2 and Table S3). These include 26 strains releasing over 10,000 μM P_i_, representing conversion of >22% of theoretically available P_i_ (see Note S2). These high levels of P_i_ hydrolysis were reproducible across replicates (see Fig. S2).

Overall, we found that 9/29 (31%) HAP and 9/29 (31%) CP enzymes were active in at least one host. In general, activity of these enzymes was the same across the three bacterial hosts. If a phytase was active in *P. simiae*, it was likely to be active in *Ralstonia* (*r* = 0.933; *P* < 0.01). None of the BPPs showed activity in any strain. The reasons for this are unclear but may reflect a requirement for other cofactors or proteins (Note S1).

Among HAPs and CPs, sequences with extremes of length (either short [HAPs, 103 to 387 amino acids; CPs, 162 to 291 amino acids] or long [HAPs, 549 to 736 amino acids; CPs, 332 to 822 amino acids]) were more likely to be inactive (see Fig. S3). Active HAPs fell within the range of 397 to 544 amino acids, while active CPs were restricted to between 293 and 331 amino acids. We considered the possibility that phytase genes deriving from bacterial lineages more closely related to our host bacterial strains were more likely to be active *in situ*. HAPs and CPs from *Proteobacteria* were slightly more likely to be active (10/22 [45%]) than phytases originating from other bacterial phyla or metagenomes (8/36 [22%]) (see Fig. S4); however, this difference was not significant (Fisher’s exact test, *P* = 0.08).

### Growth capacity of phytase-engineered strains.

When grown under P_i_-limited conditions with phytate as the only available P_i_ source, 86% of engineered *P. simiae* strains shown to have phosphate-solubilizing activity in the liquid medium grew to higher final cell densities than corresponding landing pad strains (see Table S4). Under P_i_-replete conditions, most engineered *P. simiae* strains had an extended lag phase when induced but ultimately had a similar maximum population size as the wild-type and landing pad strains (see Fig. S5). These results suggest that carrying an engineered phytase does not impose an excessive burden on the cell and may offer a growth advantage under P_i_-limited conditions.

### Localization and pH range of phytase activity.

**FIG 3:**
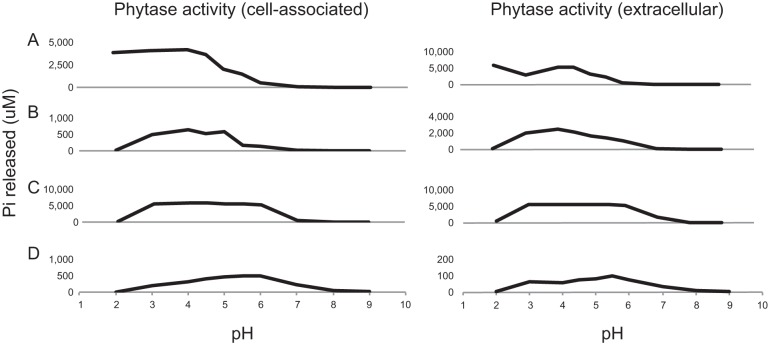
Effect of pH on phytase activity. (A to D) Phytase activity in the extracellular medium for H07 (A), H19 (B), H11 (C), and C24 (D). The values are the averages from three experiments. The tested phytases have optimal activities across a range of pHs.

To further characterize the active phytate-hydrolyzing strains, we isolated cell-associated and extracellular protein fractions from four of the constructs (three HAPs and one CP) and tested phytase activity across a range of pHs. While all were active at acidic pHs, they had different optima (from 2 to 6) (Fig. 3). The CP analyzed had higher levels of phytase activity in the cell-associated fraction than in the extracellular fraction (Fig. 3D). Results from the four HAPs were mixed: two (H07 and H19) had higher levels of phytase activity in the extracellular fraction, while one had similar levels of activity in both fractions (H11). These results suggest that phytases are exuded into the medium and exhibit different biochemical properties. This is important in generating consortia of bacteria with potentially diverse capabilities such that they complement one another under different environmental conditions.

### Plants treated with phytase-expressing bacterial strains exhibit improved growth under P_i_-limited conditions.

**FIG 4:**
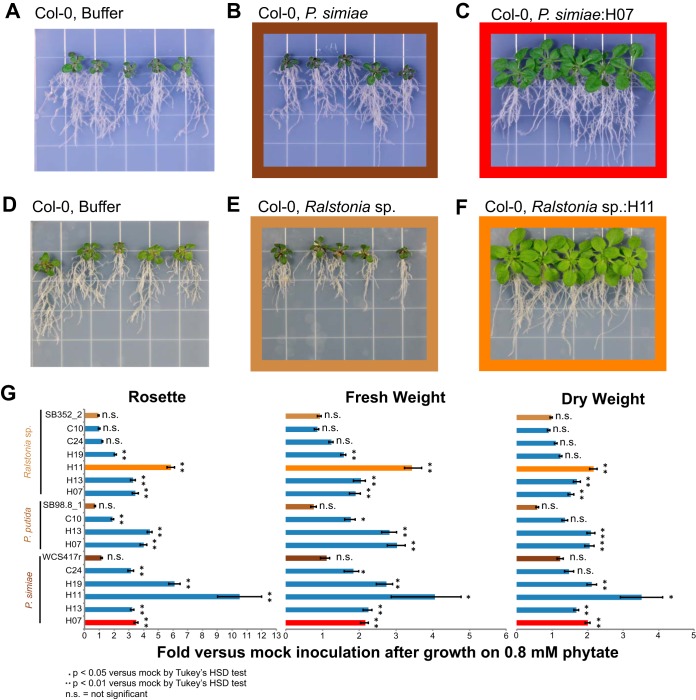
Engineered strains improve plant growth under P_i_-limited conditions. *Arabidopsis* was grown on one-half-strength Murashige and Skoog agar supplemented with 0.8 mM sodium phytate and specific bacterial treatments. (A to F) Representative images 17 days postinoculation with one-quarter-strength Ringer’s solution (A), *P. simiae* WCS417r (B), or *P. simiae*:H07 (C) or 19 days postinoculation with one-quarter-strength Ringer’s solution (D), *Ralstonia* sp. 352_2 (E), or *Ralstonia* sp:H11 (F). (G) Fold change in rosette size, fresh weight, and dry weight upon engineered and unengineered strain treatments versus mock inoculation for all strains tested. Brown bars indicate control strains without engineered phytases. Individual plant photos in B, C, E, and F are colored to correspond with data in G.

Our next aim was to determine whether these engineered strains would result in improved plant growth on phytate. The engineered strains used here include those used in the previous activity localization studies (Fig. 3). They represent the best-performing strains from the initial determination of P_i_ mobilization (Fig. 2). We used an experimental system where *Arabidopsis* is grown on agar plates containing either 1 mM P_i_ or 0.8 mM phytate. Plates were then amended with engineered bacteria, control bacteria (no phytase), or no bacteria (mock). For each condition, we used at least 25 plants grown across at least 5 replicate plates. At the end of 3 weeks, we compared plants grown under different conditions and determined whether the engineered bacterial strains provided a growth advantage to the plants (see Materials and Methods**)**. In total, 14 different host/gene combinations were tested in assays on *Arabidopsis* with phytate as the sole phosphate source. These included representatives of each host and both functional classes of phytase (HAPs and CPs) (Fig. 4).

The growth of *Arabidopsis* grown on phytate as the only phosphate source (P_i_ limited) and no added bacteria was consistently less than under P_i_-replete conditions (1 mM P_i_ added) (see Fig. S6 to S8). Plants exhibited limited growth and accumulation of anthocyanin resulting in a darker leaf color, consistent with the inability of *Arabidopsis* to access sufficient P_i_ from phytate (Fig. 4A and D).

*Arabidopsis* grown on phytate and treated with the control strains did not show increased growth benefit compared to that with the mock treatment (Fig. 4B and E; Fig. S6 to S8). These plants still have a P starvation phenotype, suggesting that the control bacteria are not releasing sufficient P_i_ from phytate to improve plant growth.

In contrast, *Arabidopsis* treated with 12/14 of the engineered strains had higher fresh weight, dry weight, and/or rosette size than the controls (Fig. 4C, F, and G), with plant growth only slightly less than under P_i_-replete conditions (Fig. S6 to S8). We consistently recovered an average of 2 × 10^8^ to 2 × 10^9^ CFU per g of root from the bacterium-treated plants tested at the conclusion of the assay, while no CFU were recovered from the mock controls (see Fig. S9). These results suggest that engineered bacteria are releasing P_i_ from phytate and that some of this is available to the plants.

We observed improved growth of *Arabidopsis* in the presence of all 10 strains that were engineered to contain HAPs (four *Ralstonia* strains, four *P. simiae* strains, and two P. putida strains, containing H07, H11, H13, or H19) (Fig. 4G). The CPs tested (C10 and C24) only improved plant growth when expressed in the pseudomonads. This is consistent with the relatively low activity of C10 and C24 *Ralstonia* strains versus that of pseudomonad strains in liquid culture assays (Fig. 2) and highlights the value of testing multiple different strains. The impact on plant growth as assessed by weight was generally correlated with absolute P_i_ mobilization (dry weight, *r* = 0.57 [*P* = 0.03]; rosette size, *r* = 0.48 [*P* = 0.08]; fresh weight, *r* = 0.60 [*P* = 0.02]).

## DISCUSSION

Phosphate-solubilizing microorganisms have potential as biological complements to phosphate fertilizers (9, 15). Here, we used a combinatorial synthetic biology approach to engineer phylogenetically diverse phytases into the genomes of three root-associated bacteria (Fig. 1). Through liquid culture assays, we identified 41 strains, with diverse pH optima, capable of hydrolyzing P_i_ from phytate (Fig. 2 and 3). The majority (12/14) of tested phytate-solubilizing strains improved the growth of *Arabidopsis* in agar plate assays with phytate as the only phosphate source (Fig. 4). Overall plant growth was generally correlated with absolute P_i_ mobilization by the strain applied. Strains releasing smaller amounts of P_i_ in liquid cultures had no significant impact on plant growth (C10 and C24 expressed in *Ralstonia*), while those that released large amounts of P_i_ in liquid culture positively impacted plant growth (e.g., H11 expressed in *Ralstonia* or *P. simiae*) (Fig. 4).

The combinatorial approach described here offers several benefits to the development of biological complements to phosphate fertilizers. First, it provides control over the bacterial host. While environmental isolates with high levels of phytase activity have been identified (35), it is often not known if these strains are also capable of colonizing plant roots. In contrast, our approach started with known root-colonizing bacteria as hosts and introduced high levels of desired phytase activity. Second, synthetic approaches enable the generation of consortia of bacteria with complementary properties. For example, in this study, we used three bacterial host species in combination with phytases that have peak activity across a range of pHs, such that one strain may always be active under diverse conditions. In the future, this approach may be expanded to harness other phosphate-solubilizing activities (for example, different enzymes or pathways which produce metabolites capable of inorganic phosphate solubilization) or other growth-promoting capabilities such as N_2_ fixation or hormone production. Finally, by directly cloning new enzymes into the genome of root-associated strains versus utilizing plasmids as has been done previously, we can circumvent plasmid loss under nonselective conditions (23).

The bacteria engineered in this study may be expected to colonize and persist in environments similar to those from which they were isolated, namely, the rhizosphere and soils. Specifically, *P. simiae* WCS417 was originally isolated from the rhizosphere of wheat (36) and subsequently shown to colonize crops such as lettuce, tomato, cucumber, and potato, where it outcompeted the indigenous microbial community (25). It additionally was successful as a biocontrol against the plant pathogen Gaeumannomyces graminis var. *tritici* (causative agent of wheat take-all disease) when applied as a seed treatment prior to planting in marine loam soil (36). This is especially relevant, as one of the failures of biocontrol of plant pathogens, and hence possibly PGPB in general, relates to the inability of the desired strains to compete with the many different microbes native to those environments (37). *P. simiae* WCS4174 has already proven itself successful in this regard. Likewise, P. putida KT2440 has been found to establish itself at high levels when applied as a seed coating in the rhizosphere of broad bean plants and corn, as well as in the surrounding bulk soils, in field trials (26) in Granada, Spain.

Bacteria that naturally hydrolyze phytate have been isolated from a number of sources, including soil (38), poultry feces (39), and river sediment (35). For example, Richardson and Hadobas (38) isolated two fluorescent pseudomonads with identical 16S rRNA gene sequences from composted garden soil. They found that these strains hydrolyzed up to 81% of phosphate contained within added phytate. The best-performing strains in this study hydrolyzed 60% of phosphate contained within added phytate. An advantage of the engineered strains is that as the bacteria are root associated, the root will likely capture a higher proportion of liberated phosphate than it could from the naturally phytate-hydrolyzing bacteria potentially found in the bulk soil.

While the approach described here represents a potential avenue to improve P_i_ availability in the rhizosphere, other challenges remain. In particular, improving the accessibility of phytate and other organophosphates sequestered on soil colloids remains an important area of research (4, 5). One approach may be to expand the engineering of root microbiota to include additional genes that may aid mobilization, such as siderophores for the solubilization of iron-phosphate complexes (40). In summary, our data provide proof of principle that DNA synthesis approaches can be used to generate plant-associated strains with novel capabilities benefitting plant growth.

## MATERIALS AND METHODS

### Identification of phylogenetically diverse phytases.

A comprehensive set of 2,518 putative phytase enzyme nucleotide and amino acid sequences was obtained by downloading all sequences annotated with pfam domains PF00328 (HAPs), PF02333 (BPPs), or PF14566 (CPs) in the Integrated Microbial Genomes and Microbiomes database (IMG/M, accessed May 2015; https://img.jgi.doe.gov/). Amino acid sequences were aligned using MAFFT (41), and a phylogenetic tree was constructed using FastTree (42). To extract a highly informative set of representatives that cover maximal phylogenetic distance, we used the MaxPD algorithm as described in reference 32. This yielded a set of 96 phytase representatives for synthesis. Due to failure at various subsequent steps, this was reduced to a final set of 82 phytases for evaluation.

### Phytase gene synthesis and cloning.

Amino acid sequences were codon optimized for expression in *P. simiae* using BOOST (33). Assembled products were cloned into pW26 vector by In-Fusion cloning (Clontech) in TransforMax EC100D *pir*^+^ cells (Epicentre). Each plasmid was then transformed into Escherichia coli WM3064, a diaminopimelic acid (DAP) auxotroph, in preparation for conjugation.

### Transformation of root-associated bacteria.

The plant root-associated strains *P. simiae* WCS417r:pw17, *Ralstonia* sp. UNC404CL21Col:SB352_2, and P. putida KT2440:SB98_8.1 were selected as hosts for phytase-solubilizing genes. Each of these strains was previously engineered with a genomic insertion of a *lox* targeting cassette (a “landing pad”) to facilitate easier genomic integration of other genes and pathways (27). To create the engineered phytase-containing strains, each host strain was conjugated with E. coli WM3064::pw26-phytase. After 6 to 8 h of growth on LB plus DAP, conjugations were resuspended in 2 ml LB, and 100 μl of a 10^−3^ dilution of this LB was plated on LB-agar plates with apramycin to isolate single colonies. After 36 h of growth at 28°C, individual colonies were streaked out on LB-kanamycin and LB-apramycin to identify apramycin-resistant kanamycin-sensitive colonies. These colonies were additionally verified for proper integration by colony PCR using primers that hit within the landing pad close to the site of gene insertion (primer pair: 5′-TCCCGCGAAATTAATACGAC-3′ and 5′-CAGCCAACTCAGCTTCCTTT-3′).

### Bacterial strains, growth, and inoculation.

All bacterial strains were routinely cultured in LB broth (Lennox, L7658; Sigma-Aldrich, St. Louis, MO) or LB Miller broth (MBLE-7030; GrowCells, Irvine, CA) supplemented with the appropriate antibiotic at 28°C in a shaking incubator at 250 rpm. For plant assays, cultures were grown for approximately 4 to 7 h depending on the strain until the culture reached mid- to late log phase (optical density at 600 nm [OD_600_] of 0.4 to 0.7). Media were supplemented with apramycin (SB352_2, 1,000 μg/ml; pw17, 50 μg/ml; SB98_8.1, 50 μg/ml) or kanamycin (500 μg/ml) as required. Cells were then harvested by centrifugation (4,000 rpm for 5 min) and washed 2 times by resuspension in 10 ml of one-quarter-strength Ringer’s solution (Sigma-Aldrich). After being washed, the cells were resuspended in 10 ml of one-quarter-strength Ringer’s solution, and the OD of the resuspension was calculated by spectrophotometry (using a 1:10 dilution).

Experiments to study the growth parameters of *P. simiae* WCS417r, *P. simiae* WCS417r:pw17, and the engineered phytase-containing derivates were carried out in LB broth containing the appropriate antibiotic and 1 mM IPTG in 96-well plates. Optical density was determined at 600 nm on a Tecan Infinite 200 Pro plate reader. Data were analyzed using the R package growthcurver (v0.2.0).

### Liquid culture assays for phytase activity and tricalcium phosphate solubilization.

To assess phytase activity, cells of each engineered strain were grown in phytase-specific medium (PSM) [1.5% glucose, 0.5% (NH_4_)_2_SO_4_, 0.05% KCl, 0.01% MgSO_4_·7H_2_O, 0.01% NaCl, 0.01% CaCl_2_·2H_2_O, 0.001% FeSO_4_, 0.001% MnSO_4_, pH 6.5, and 0.5% sodium phytate [39]), and the supernates were collected at various time points. To assess tricalcium phosphate solubilization as a proxy for organic acid production, cells of each landing pad strain were grown in National Botanical Research Institute’s phosphate growth medium (NBRIP) with a minor modification [1% glucose, 0.5% MgCl_2_·6H_2_O, 0.025% MgSO_4_·7H_2_O, 0.02% KCl, 0.01% (NH_4_)_2_SO_4_, 0.4% Ca_3_(PO_4_)_2_ (43)], and the supernatants were collected after 1 to 3 days of growth. The release of P_i_ from phytate or NBRIP was monitored using the QuantiChrom phosphate assay kit (Bioassay Systems, Hayward, CA) across 3 (phytate) or 2 (NBRIP) technical replicates in 96-well plates at 600 nm on a Tecan Infinite 200 Pro plate reader.

### Phytase enzyme assays.

All assays were conducted using freshly prepared cultures. Starter cultures of pw17::phytase were grown overnight and used to inoculate a flask (250 μl of culture into 25 ml of LB medium) containing 50 μg/ml apramycin and 1 mM IPTG. After 46 h of growth, crude supernatant and cells were separated by centrifugation (4,000 × *g* for 15 min at 4°C). The supernatant and cell pellet were then separately assayed for phytase activity.

Fifteen milliliters of the supernatant was transferred to an Amicon Ultra-15 centrifugal filter unit (MilliporeSigma) and subjected to centrifugation at 4,000 × *g* for 30 min. The flowthrough was discarded, and the remaining supernatant was applied to the filter unit and again subjected to centrifugation. The flowthrough was discarded, and the concentrated proteins were resuspended with 14 ml of 1 M Tris-HCl (pH 7) and subsequently subjected to a third centrifugation. The concentrated proteins were then transferred to a new tube, the volume was brought up to 2 ml with 1 M Tris-HCl (pH 7), and protein concentration was measured using the Quick Start Bradford protein assay kit (Bio-Rad Laboratories).

The culture cell pellet was suspended in lysis buffer (50 mM HEPES, 300 mM NaCl, 10 mM imidazole, pH 8), 0.8 mg ml^−1^ lysozyme and 72.5 U Benzonase nuclease were added, and the mixture was incubated for 15 to 30 min on ice. The lysate was then subjected to centrifugation at 12,000 × *g* for 15 to 30 min at 4°C. The resulting supernatant was decanted into an Amicon Ultra-4 centrifugal filter unit (MilliporeSigma) and centrifuged at 4,000 × *g* for 30 min. The flowthrough was discarded, and concentrated proteins were resuspended within the filter unit in 2 ml Tris-HCl (pH 7) and subsequently subjected to a second centrifugation. The concentrated proteins were then transferred to a new tube, the volume was brought up to 2 ml with 1 M Tris-HCl (pH 7), and protein concentration was measured using the Quick Start Bradford protein assay kit (Bio-Rad Laboratories).

All enzymes were assayed using sodium phytate (5 mM) as a substrate at 37°C against a control without the enzyme source in the reaction mixture. Phytase pH optima were determined using the buffers as described in reference 44 at 0.1 M with slight modifications: glycine-HCl (pH 2 to 3), sodium acetate-HCl (pH 4 and 5), sodium acetate (pH 4.5), MES (morpholineethanesulfonic acid)-Tris-HCl (pH 5.5 to 6), Tris-HCl (pH 7 to 8), and glycine-NaOH (pH 9). Briefly, 70 μl of buffer/substrate mixture was added to 4 μg of enzyme preparation (in a 30-μl volume) in a 96-well plate. After 30 min of incubation, the reaction was stopped with 100 μl of 15% trichloroacetic acid, and the concentration of liberated P_i_ was determined at 600 nm on a Tecan Infinite 200 Pro plate reader.

### Plant growth assays.

All plants used in this study were Arabidopsis thaliana ecotype Columbia (Col-0). Prior to growth, seeds were surface sterilized by immersion in 70% ethanol for 5 min, followed by 10 min in 50% bleach plus 0.1% Triton X-100 and several rinses in sterile water. Seeds were germinated in 24-well plates containing one-half-strength Murashige and Skoog (MS) medium with no phosphate and 0.5% sucrose. Seven to ten days after planting, 5 seedlings were transplanted to each square petri dish containing 0.5× MS medium with no sucrose, either 0.8 mM phytate or 1 mM P_i_ (KH_2_PO_4_) as the sole phosphate source, 10 mM 3-(*N*-morpholino)propanesulfonic acid (MOPS), an IPTG overlay (48 μl of 100 mM IPTG spread over the 10-cm^2^ petri dish), and 1% Noble agar, adjusted to pH 7.0, and inoculated with 100 μl of either one-quarter-strength Ringer’s solution or bacteria diluted to an *A*_600_ of 0.125 in one-quarter-strength Ringer’s solution using sterile glass beads. Each treatment, including controls, was replicated on 5 plates, for a total of 25 plants per treatment. Seventeen to 21 days after inoculation, plants were imaged and then removed from plates, and fresh and dry weights were determined as described in reference 45, with some modifications. Briefly, roots were placed onto tared aluminum weigh boats and weighed on a laboratory scale (MS105; Mettler Toledo, Columbus, OH) to determine fresh weight. Samples were then dried in a gravity convection oven at 75 ± 2°C for 16 h, and dry weight was determined on the same laboratory scale. Rosette sizes were calculated using the software ImageJ (v1.50i; http://imagej.nih.gov/ij). Statistical analyses of rosette size, fresh weight, and dry weight were performed using analysis of variance (ANOVA) and Tukey’s honestly significant difference method within the R software package (46). Statistical analysis for Fig. S5 to S7 in the supplemental material was performed using a *t* test within Excel. Plant images used for rosette size estimation are available at the Open Science Framework database: https://osf.io/m6gde/. These files can be accessed directly by downloading them from the OSF server. Roots were washed six times to remove bacteria not attached to the root and then bead beaten; the resulting lysate was serially diluted to determine CFU.

## Supplementary Material

Supplemental file 1

Supplemental file 2

Supplemental file 3
