# Supplementary material for: Engineered Root Bacteria Release Plant-Available Phosphate from Phytate
Source: Appl Environ Microbiol. 2019 Aug 29;85(18):e01210-19. doi: 10.1128/AEM.01210-19 (PMC6715853; doi:10.1128/AEM.01210-19)
Supplement: Supplemental file 1 [file AEM.01210-19-s0001.pdf]

Supplementary Tables

Supplementary Table 1. Potential pathways for Pi mobilization.

**Pathway**	**Genes**	**Function**	**Example Organism(s)**	**Reference**
Gluconate dehydrogenase	*gad*	2-ketogluconic acid production	*Pseudomonas putida* KT 2440	(1)
Pyrroloquinoline quinone (PQQ) synthase genes	*pqqFABCDEG*	Cofactor necessary for assembly of the glucose dehydrogenase holoenzyme	*Erwinia herbicola, Pseudomonas* spp.	(2-4)
Pathway unknown	*gabY*	Gluconic acid production	*Burkholderia cepacia*	(5)
Glucose dehydrogenase	*gcd*	Gluconic acid production	*Escherichia coli*	(5)
Bacterial nonspecific acid phosphatases	*acpA*, *phoC*, *napA, napD, napE*	Release Pi from organic phosphates	*Francisella tularensis, Morganella morganii, Rhizobium meliloti*	Reviewed in (6)
Phytases	*phyA*, *phy, appA, appA2*	Release Pi from phytate	*Aspergillus niger, Bacillus* spp, *E. coli*	(7-9)
C-P lyase reaction	*phnCDEFGHIJKLMNOP*	Releases Pi from alkyl-phosphonates	*Escherichia coli*	(10)
Phosphite-specific oxidoreductase	*ptxD*	Produces Pi from phosphite	*Pseudomonas stutzeri* WM88	(11, 12)
Enterobactin	*entABCDEF*	Production of the siderophore enterobactin	*Escherichia coli*	Reviewed in (13)

Supplementary Table 2: 82 evaluated phytases. Localization results based on PSORTb v. 3.0.2.

Supplementary Table 3: Overview: Average free phosphate levels in culture supernatant where phytate is the only phosphate source over time for each construct expressed in *P. simiae*, *Ralstonia* sp., and *P. putida*. Additional worksheets contain raw data for each replicate. See materials and methods for a description of how these measurements were taken. N.D. = no data.

Supplementary Table 4. Optical densities of selected phytase engineered strains as well as the landing pad strains at day 10 (*P. simiae* and *P. putida*) or day 6 (*Ralstonia* sp.) in culture where phytate is the only phosphate source (Phytase Specific Medium).

**Strain**	**OD_{600}**
P. simiae pw17	0.12
P. simiae:C04	0.12
P. simiae:C05	0.13
***P. simiae*:C08**	***0.64*****
P. simiae:C09	0.13
***P. simiae*:C10[a]**	**0.96*****
P. simiae:C11	0.13
P. simiae:C12	0.13
P. simiae:C13	0.06***
P. simiae:C14	0.13
***P. simiae*:C19**	**0.10**
***P. simiae*:C21**	**0.91*****
***P. simiae*:C23**	**0.39****
***P. simiae*:C24[a]**	**0.89*****
P. simiae:C25	0.12
P. simiae:C26	0.07***
P. simiae:C29	0.11
***P. simiae*:H07[a]**	**0.84*****
P. simiae:H10	0.09**
***P. simiae*:H11[a]**	**0.79****
***P. simiae*:H13**	**0.95*****
P. simiae:H14	0.06***
***P. simiae*:H15**	**0.75*****
P. simiae:H16	0.16**
P. simiae:H18	0.13
***P. simiae*:H19[a]**	**0.10***
P. simiae:H23	0.07***
***P. simiae*:H24**	**0.76*****
P. simiae:H27	0.22**
P. putida SB98_8.1	0.13
***P. putida*:C10[a]**	**0.85***
***P. putida*:C24**	**0.86****
***P. putida*:H07[a]**	**0.58****
***P. putida*:H11**	**0.56****
***P. putida*:H13[a]**	**0.45****

***P. putida*:H19**	**0.90*** **
Ralstonia sp. SB352_2	0.23
Ralstonia sp.:C10	0.23
Ralstonia sp.:C24	0.23
***Ralstonia* sp.:H07**	**0.26****
***Ralstonia* sp.:H11**	**0.51*****
***Ralstonia* sp.:H13**	**0.26***
***Ralstonia* sp.:H19**	**0.29**

Results are expressed as the mean of at least three replicates. Each OD_{600} reading is compared with that of the corresponding landing pad strain by Student's t-test. Strains in bold release ≥10,000 uM Pi from phytate after 10 d in Phytase Specific Medium.

*$P<0.05$

**$P<0.01$

***$P<0.001$

[a]Tested on plants

Supplementary Figures

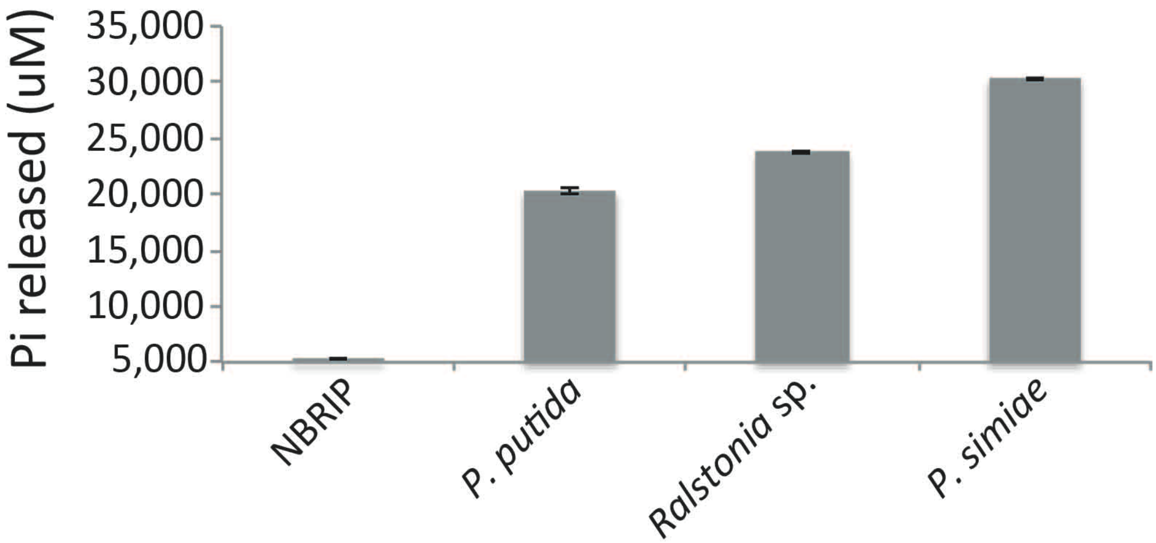

Supplementary Figure 1. Free phosphate levels in culture supernate where tricalcium phosphate is the only phosphate source at 3 days for the NBRIP media control, *P. putida* SB98_8.1, and *Ralstonia* sp. SB352_2, and 1 day for *P. simiae* WCS417r. PGPB were screened for the ability to release Pi from tricalcium phosphate using the malachite green-based QuantiChrom Phosphate Assay Kit (BioAssay Systems); *n* = 2 replicates; averages ± standard deviation are shown.

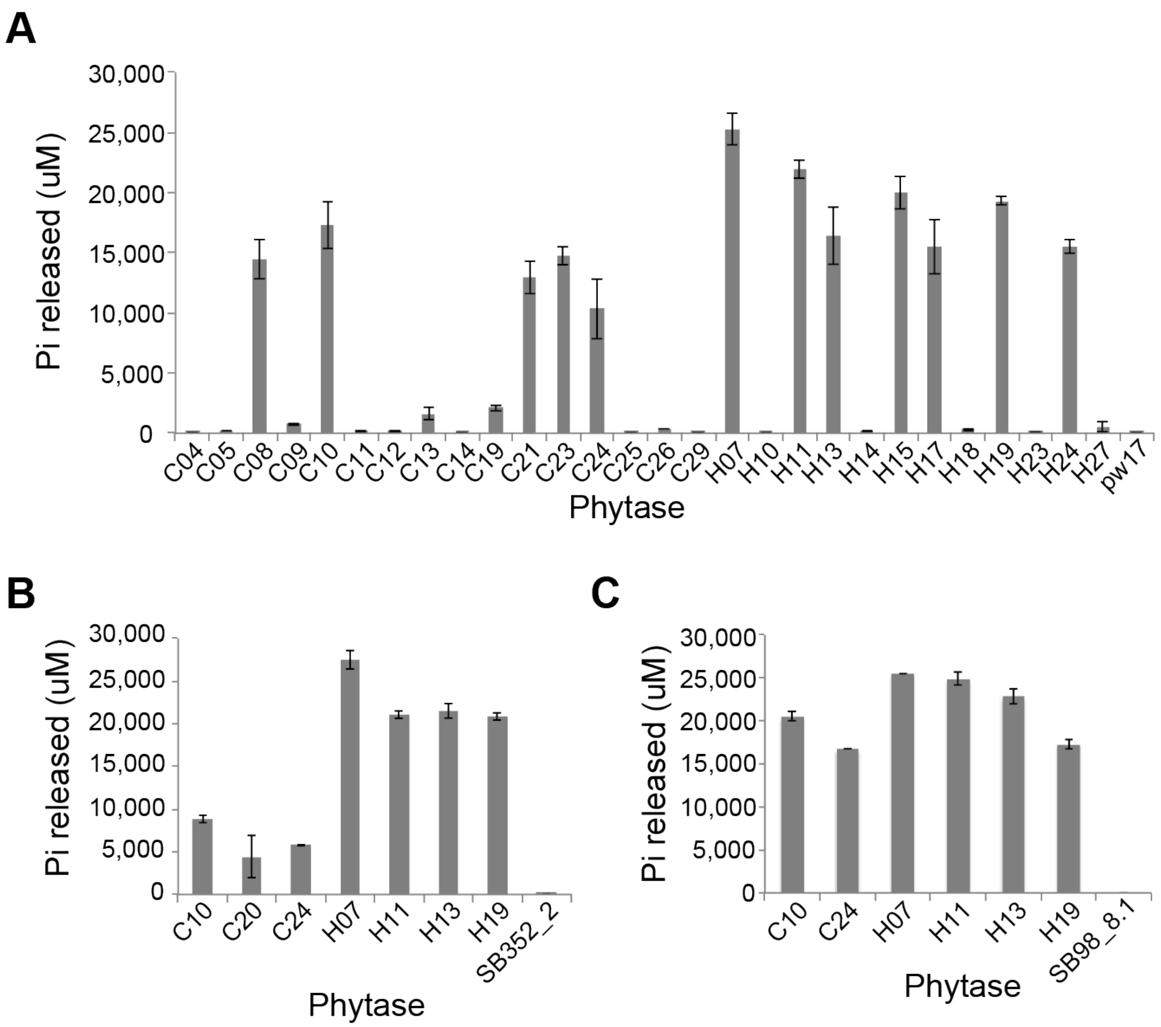

Supplementary Figure 2. Free phosphate levels in culture supernate where phytate is the only phosphate source at 10 days for constructs expressed in (A) *P. simiae* WCS417r:pw17, (B) *Ralstonia* sp. SB352_2, and (C) *P. putida* SB98_8.1. PGPB with engineered phytases were screened for the ability to release Pi from phytate using the malachite green-based QuantiChrom Phosphate Assay Kit (BioAssay Systems); $n \geq 3$ replicates; averages ± standard deviation are shown.

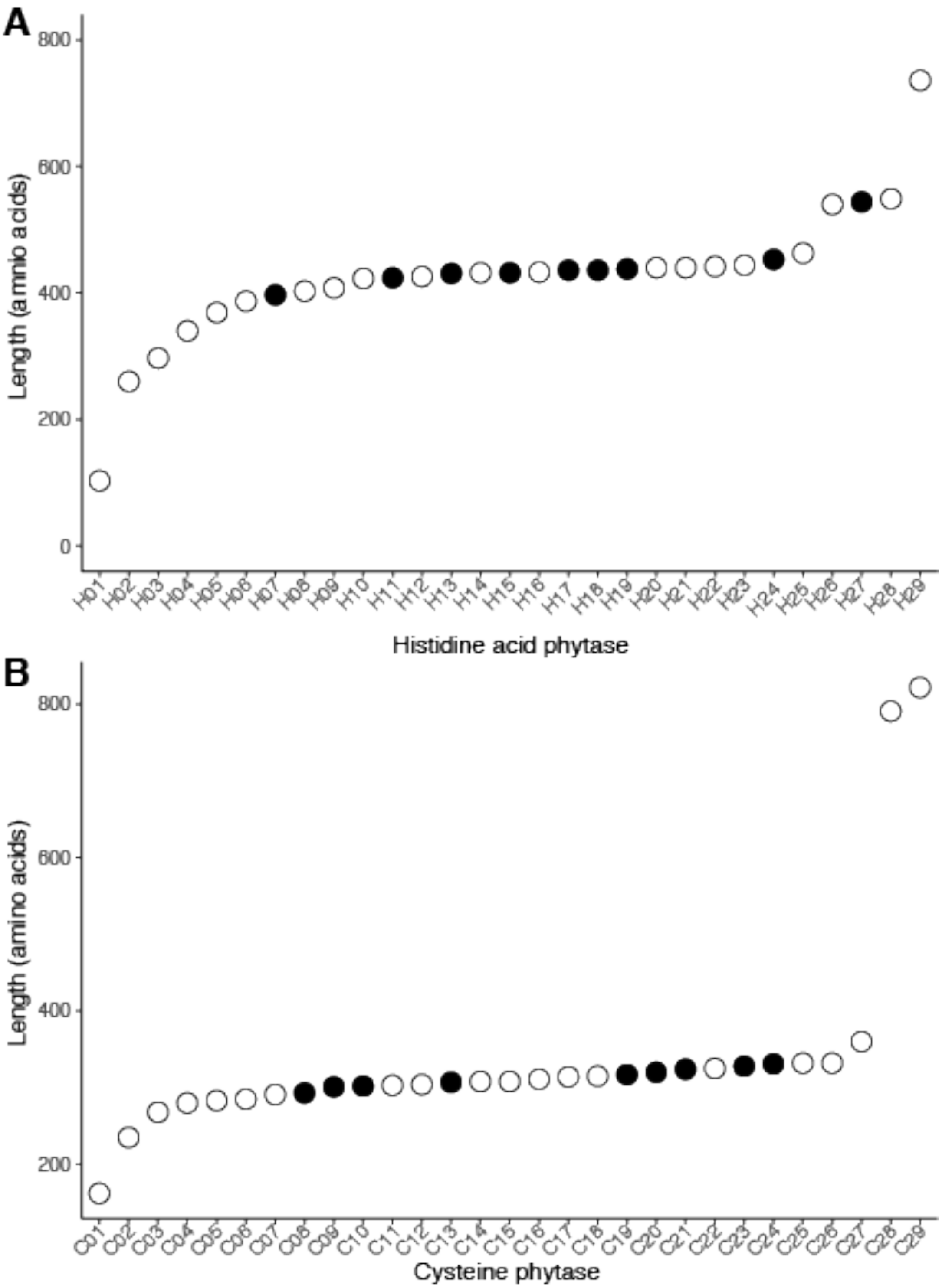

Supplementary Figure 3. Amino acid length versus activity of synthesized phytases. (A) Histidine acid phytases. (B) Cysteine phytases. For both, filled circles denote active phytases and open circles denote phytases found to be inactive.

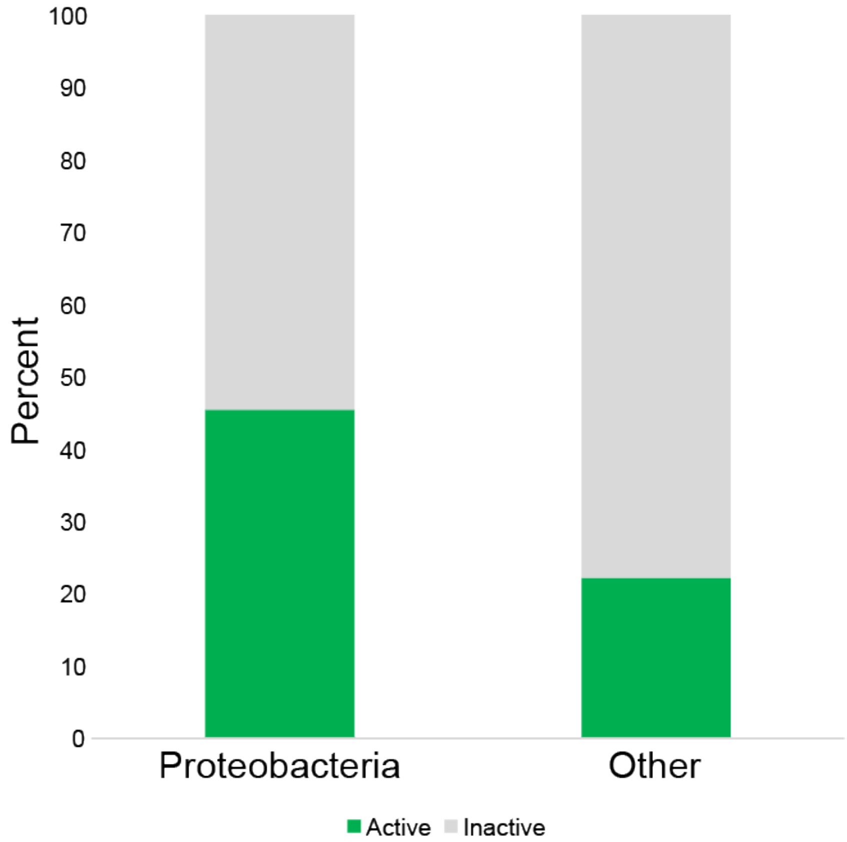

Supplementary Figure 4. Phylum-level classification of source of phytase enzymes versus their activity in the *Proteobacteria* hosts.

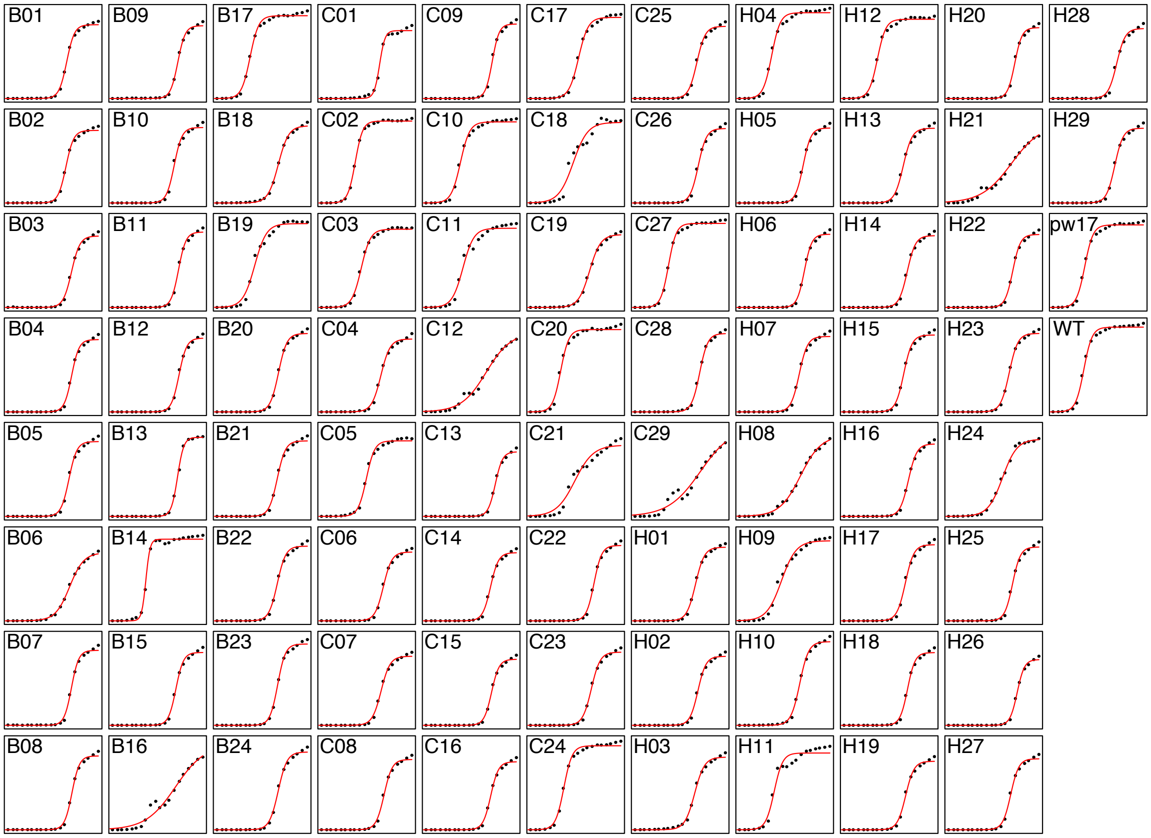

Supplementary Figure 5. Growth profile of *P. simiae* WCS417r, the landing pad strain (pw17), and its engineered phytase-containing derivatives in LB medium over 35.5 h. Red lines represent best fit logistic curves. OD_{600nm} value at each time point is the mean of three replicates. Most induced engineered strains have longer lag phases but similar maximum population size to wild type and landing pad strains.

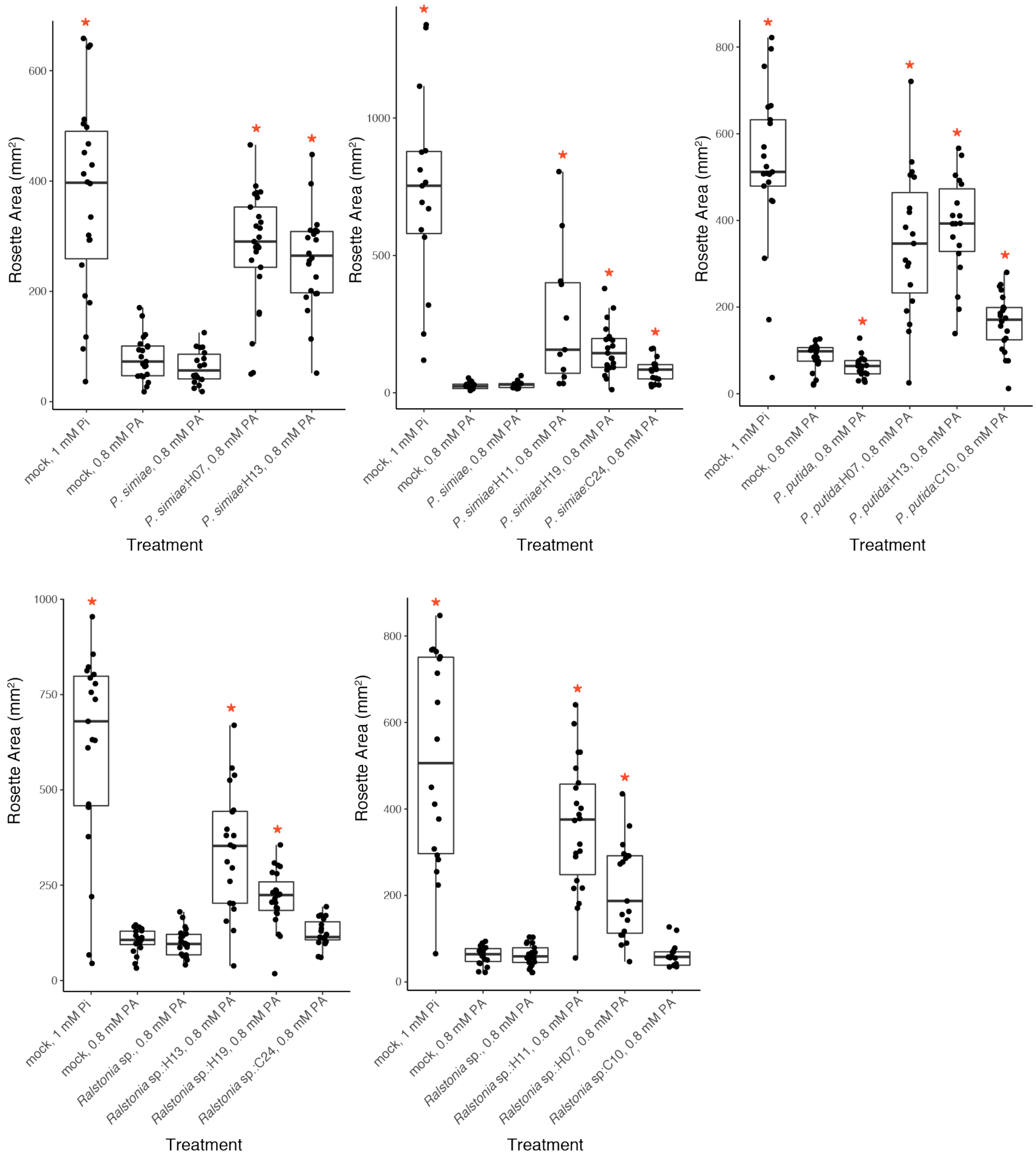

Supplementary Figure 6. Effect of engineered strains on *Arabidopsis* rosette size. Plants were grown on plates with either full Pi (1 uM) or 0.8 mM phytic acid (PA) and inoculated with one of the strains engineered in this study or a mock inoculation of Ringer’s solution. Each panel represents a separate experiment. **P* < 0.05 versus mock, 0.8 mM PA by two-sided *t*-test.

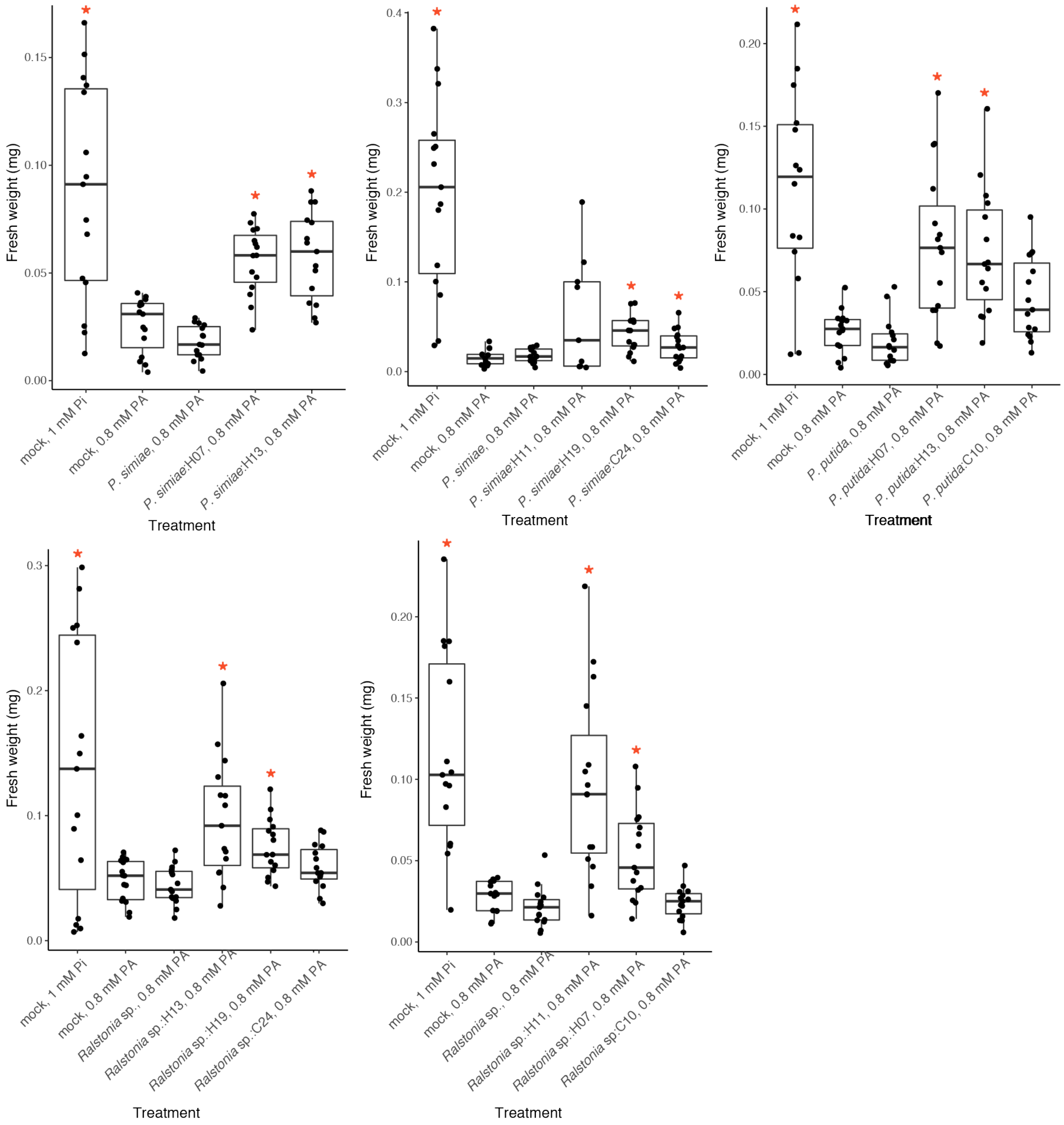

Supplementary Figure 7. Effect of engineered strains on *Arabidopsis* fresh weight. Plants were grown on plates with either full Pi (1 uM) or 0.8 mM phytic acid (PA) and inoculated with one of the strains engineered in this study or a mock inoculation of Ringer's solution. Each panel represents a separate experiment. **P* < 0.05 versus mock, 0.8 mM PA by two-sided *t*-test.

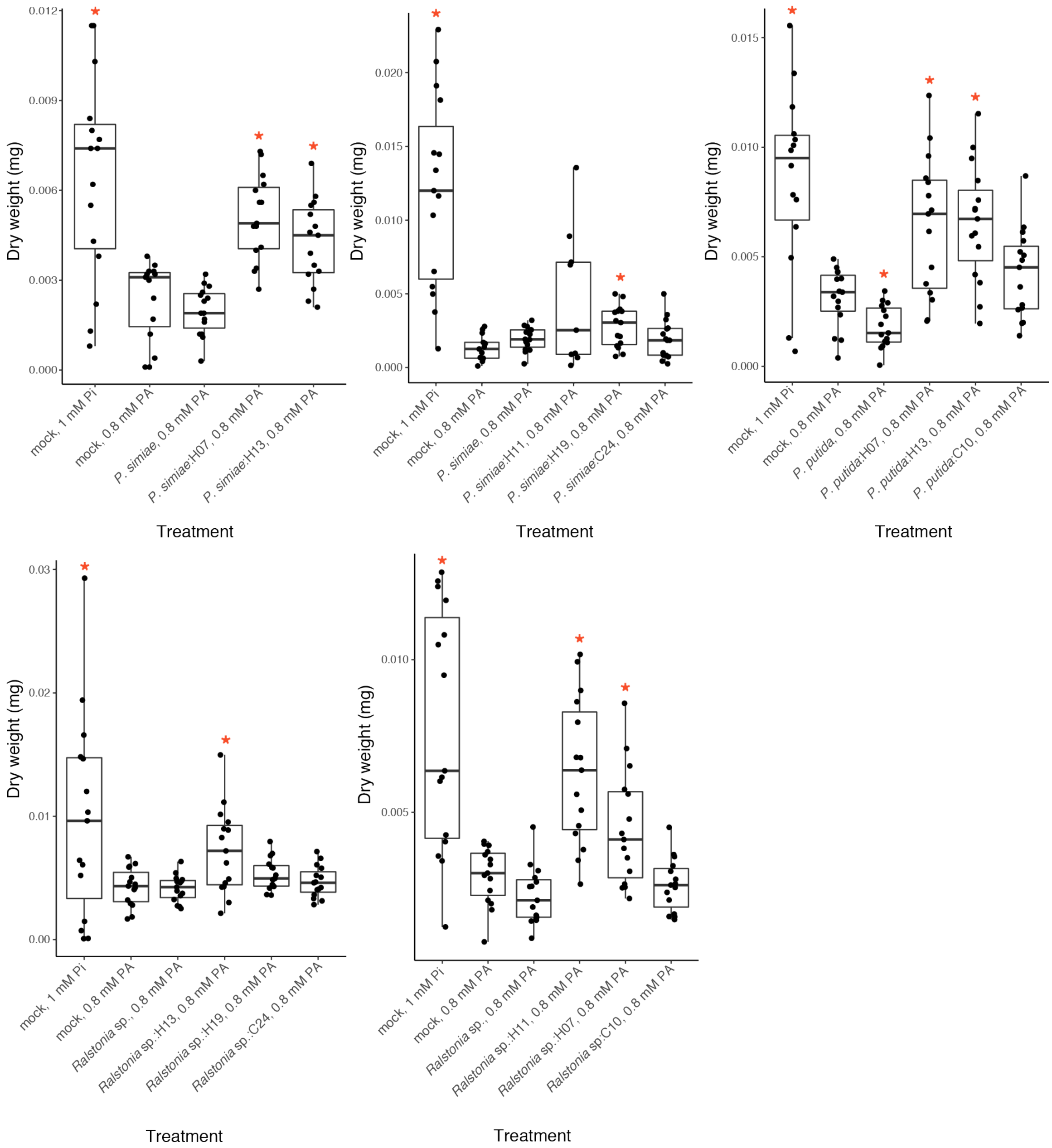

Supplementary Figure 8. Effect of engineered strains on *Arabidopsis* dry weight. Plants were grown on plates with either full Pi (1 uM) or 0.8 mM phytic acid (PA) and inoculated with one of the strains engineered in this study or a mock inoculation of Ringer's solution. Each panel represents a separate experiment. *$P < 0.05$ versus mock, 0.8 mM PA by two-sided *t*-test.

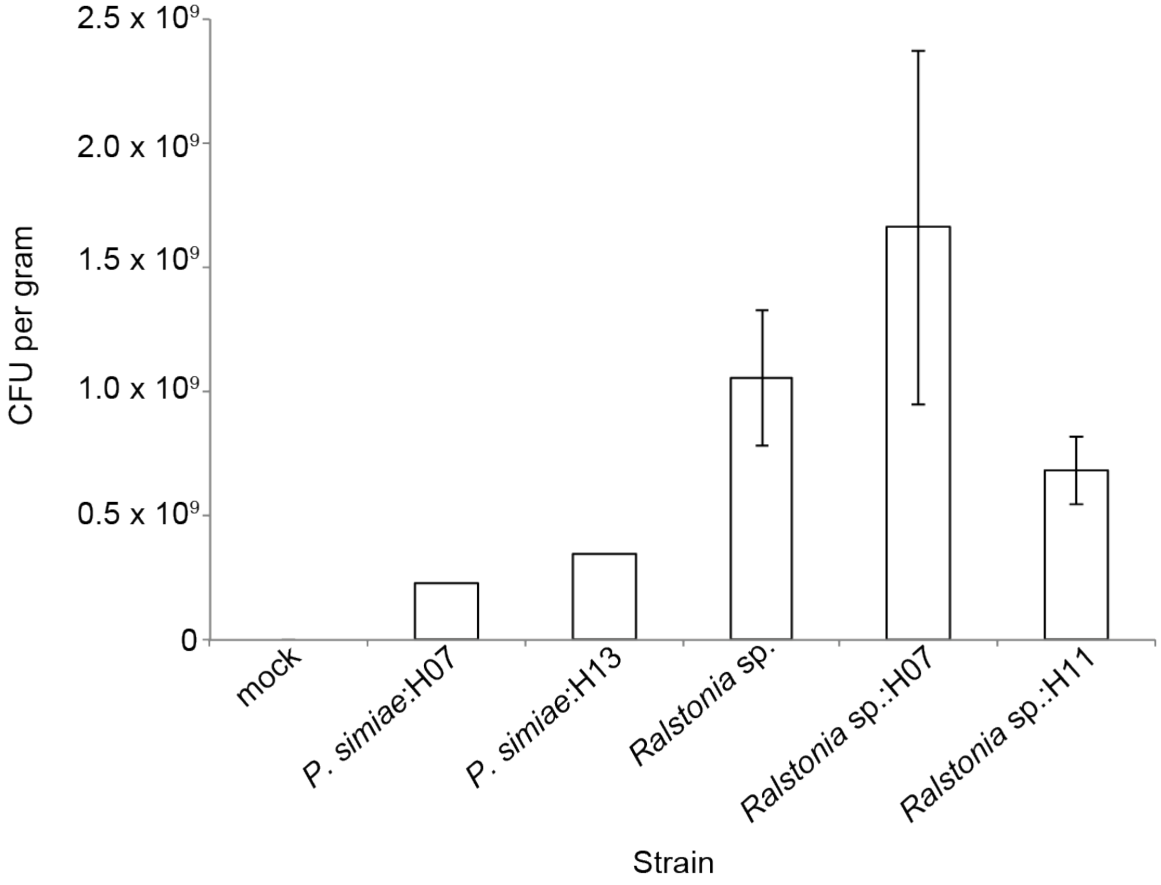

Supplementary Figure 9. CFUs attached to roots at end of plant assay. Plants were grown on plates with 0.8 mM phytate and inoculated with an engineered strain or a mock inoculation of Ringer's solution. Root lysate was serially diluted to determine CFUs (see Methods).

Supplementary Note 1

It has been noted that BPPs are often found in the genome in an operon with a TonB-dependent receptor-like gene (14). Interestingly, the *P. simiae* strain used in this study fact has a native BPP-like gene (IMG Gene ID 2586031829) and an adjacent TonB-dependent receptor-like gene (IMG Gene ID 2586031830), however the landing pad strain did not release a notable amount of Pi (8 uM). It is also possible that the genes are not expressed under the conditions used in this study, although the BPP in *Shewanella oneidensis* has been shown to be expressed under conditions of Pi deficiency such as those used here (15). Lastly, it is known that BPPs require Ca^{2+} as a cofactor, and in fact can only hydrolyze a calcium-phytate complex, and excess free phytate can act as an enzyme inhibitor(16). Although the Phytase Specific Medium (PSM; see **Methods**) used in this assay contains Ca^{2+}, the phytate was provided as phytic acid sodium salt hydrate, which may explain the lack of activity from the BPPs observed in this study.

Supplementary Note 2

HAPs and CPs are both known to hydrolyze 5 of 6 phosphate groups (17, 18), so a release of 10,000 µM Pi represents a release of 26% theoretical Pi.

References

1. Kumar C, Yadav K, Archana G, Kumar GN. 2013. 2-Ketogluconic acid secretion by incorporation of *Pseudomonas putida* KT 2440 gluconate dehydrogenase (*gad*) operon in *Enterobacter asburiae* PSI3 improves mineral phosphate solubilization. Curr Microbiol 67:388-394.
2. Rodriguez H, Gonzalez T, Selman G. 2000. Expression of a mineral phosphate solubilizing gene from *Erwinia herbicola* in two rhizobacterial strains. J Biotechnol 84:155-161.
3. Liu ST, Lee LY, Tai CY, Hung CH, Chang YS, Wolfram JH, Rogers R, Goldstein AH. 1992. Cloning of an *Erwinia herbicola* gene necessary for gluconic acid production and enhanced mineral phosphate solubilization in *Escherichia coli* HB101: nucleotide sequence and probable involvement in biosynthesis of the coenzyme pyrroloquinoline quinone. J Bacteriol 174:5814-5819.
4. Miller SH, Browne P, Prigent-Combaret C, Combes-Meynet E, Morrissey JP, O'Gara F. 2010. Biochemical and genomic comparison of inorganic phosphate solubilization in *Pseudomonas* species. Environ Microbiol Rep 2:403-411.
5. Babu-Khan S, Yeo TC, Martin WL, Duron MR, Rogers RD, Goldstein AH. 1995. Cloning of a mineral phosphate-solubilizing gene from *Pseudomonas cepacia*. Appl Environ Microbiol 61:972-978.
6. Rodriguez H, Fraga R, Gonzalez T, Bashan Y. 2006. Genetics of phosphate solubilization and its potential applications for improving plant growth-promoting bacteria. Plant Soil 287:15-21.
7. Golovan S, Wang GR, Zhang J, Forsberg CW. 2000. Characterization and overproduction of the *Escherichia coli appA* encoded bifunctional enzyme that exhibits both phytase and acid phosphatase activities. Can J Microbiol 46:59-71.
8. Richardson AE, Hadobas PA, Hayes JE. 2001. Extracellular secretion of *Aspergillus* phytase from *Arabidopsis* roots enables plants to obtain phosphorus from phytate. Plant J 25:641-649.
9. Kim YO, Lee JK, Kim HK, Yu JH, Oh TK. 1998. Cloning of the thermostable phytase gene (phy) from *Bacillus* sp. DS11 and its overexpression in *Escherichia coli*. FEMS Microbiol Lett 162:185-191.
10. Kamat SS, Williams HJ, Raushel FM. 2011. Intermediates in the transformation of phosphonates to phosphate by bacteria. Nature 480:570-573.
11. Lopez-Arredondo DL, Herrera-Estrella L. 2012. Engineering phosphorus metabolism in plants to produce a dual fertilization and weed control system. Nat Biotechnol 30:889-U123.
12. Costas AMG, White AK, Metcalf WW. 2001. Purification and characterization of a novel phosphorus-oxidizing enzyme from *Pseudomonas stutzeri* WM88. J Biol Chem 276:17429-17436.

13. Sattely ES, Fischbach MA, Walsh CT. 2008. Total biosynthesis: in vitro reconstitution of polyketide and nonribosomal peptide pathways. Natural Product Reports 25:757-793.
14. Lim BL, Yeung P, Cheng C, Hill JE. 2007. Distribution and diversity of phytate-mineralizing bacteria. ISME J 1:321-330.
15. Cheng CW, Lim BL. 2006. Beta-propeller phytases in the aquatic environment. Arch Microbiol 185:1-13.
16. Oh BC, Chang BS, Park KH, Ha NC, Kim HK, Oh BH, Oh TK. 2001. Calcium-dependent catalytic activity of a novel phytase from *Bacillus amyloliquefaciens* DS11. Biochemistry 40:9669-9676.
17. Cosgrove DJ. 1970. Inositol phosphate phosphatases of microbiological origin. Inositol phosphate intermediates in the dephosphorylation of the hexaphosphates of *myo*-inositol, *scyllo*-inositol, and D-*chiro*-inositol by a bacterial (*Pseudomonas* sp.) phytase. Aust J Biol Sci 23:1207-&.
18. Chu HM, Guo RT, Lin TW, Chou CC, Shr HL, Lai HL, Tang TY, Cheng KJ, Selinger BL, Wang AHJ. 2004. Structures of *Selenomonas ruminantium* phytase in complex with persulfated phytate: DSP phytase fold and mechanism for sequential substrate hydrolysis. Structure 12:2015-2024.
